# Identification of conserved RNA secondary structures at influenza B and C splice sites reveals similarities and differences between influenza A, B, and C

**DOI:** 10.1186/1756-0500-7-22

**Published:** 2014-01-09

**Authors:** Lumbini I Dela-Moss, Walter N Moss, Douglas H Turner

**Affiliations:** 1 Department of Chemistry and Center for RNA Biology, University of Rochester, Rochester, New York 14627-0216, USA

**Keywords:** Influenza, RNA, Secondary structure, Splice sites, Bioinformatics, Splicing

## Abstract

**Background:**

Influenza B and C are single-stranded RNA viruses that cause yearly epidemics and infections. Knowledge of RNA secondary structure generated by influenza B and C will be helpful in further understanding the role of RNA structure in the progression of influenza infection.

**Findings:**

All available protein-coding sequences for influenza B and C were analyzed for regions with high potential for functional RNA secondary structure. On the basis of conserved RNA secondary structure with predicted high thermodynamic stability, putative structures were identified that contain splice sites in segment 8 of influenza B and segments 6 and 7 of influenza C. The sequence in segment 6 also contains three unused AUG start codon sites that are sequestered within a hairpin structure.

**Conclusions:**

When added to previous studies on influenza A, the results suggest that influenza splicing may share common structural strategies for regulation of splicing. In particular, influenza 3′ splice sites are predicted to form secondary structures that can switch conformation to regulate splicing. Thus, these RNA structures present attractive targets for therapeutics aimed at targeting one or the other conformation.

## Findings

### Background

Influenza virus causes more than 200,000 hospitalizations and about 3000 – 49,000 deaths per year in the United States alone [1,2]. Influenza A, B, and C viruses belong to the family *Orthomyxoviridae* and are characterized by segmented, single-stranded, negative-sense (−) RNA genomes. These viruses share a common ancestry but are also genetically distant, such that segment reassortment does not occur between each group [3]. Each of the (−) RNA segments is used as a template to produce two types of positive-sense (+) RNA with distinct functions: mRNA for protein production and complementary RNA (cRNA) for viral replication. Influenza B has eight genome segments that encode at least eleven proteins and influenza C has seven genome segments that encode at least nine proteins. Influenza A infects avian, human, swine, and many other mammalian species, whereas influenza B and C infect primarily humans [4-6]. Influenza B and C do not undergo pandemic-causing antigenic shifts (reassortment of segments from different subtypes) like influenza A, because both viruses contain only one antigenic subtype and have limited host specificity [7,8]. All influenza viruses are able to undergo antigenic drift, which occurs as a result of accumulation of mutations in the antigenic sites [3,7,8]. Concern, however, has been growing, as two lineages of influenza B (Yamagata and Victoria) have been co-circulating in the human population [7,9]. This has led to a novel formulation of a quadrivalent vaccine: against two strains of influenza A and two strains of influenza B [10,11], rather than the previous trivalent vaccine.

RNA structure plays important roles in many viruses. For example, internal ribosome-entry sites (IRES) in viral mRNAs are heavily structured regions, which initiate cap-independent translation by directly binding to the ribosome [12,13]. RNA structure is also used for start codon selection and viral replication [14], for packaging signals [15], for RNA editing [16], and for many more functions. RNA secondary structure also plays an important role in viral mRNA splicing regulation [17-19]. A relatively rare type of RNA structure, pseudoknots, often plays important roles in biology [20-22]. In particular, pseudoknots are important in the regulation of viral gene expression and genome replication [23,24].

RNA structure is also important in influenza. The 5′ and 3′ ends of each genome segment of influenza A, B, and C are highly conserved, partially complementary, and base pair to form a promoter region that can be either in a panhandle or corkscrew conformation [25-27]. This structure is essential in vRNA transcription, replication, and viral packaging [28-30].

A variety of de novo methods exist to predict conserved secondary structure in genomes [31-35]. RNA structure can occur in protein coding regions and has many potential functional roles [34,36]. A survey for conserved secondary structure in the (+) and (−) RNAs of influenza A was carried out [37] by scanning for thermodynamically stable and conserved regions with the program RNAz [38-40] and coupling this with evidence of suppression of synonymous codon usage (SSCU), which identifies possible constraints of secondary structure acting on codon diversity [34,41,42]. Twenty conserved, thermodynamically stable regions were identified. Secondary structure is strongly favored in the (+) RNA. Of these predicted regions, five occur at or near functionally relevant sites [37]. Two of these, occurring in the segment 8 (+) RNA, were previously proposed [43-45].

This paper extends the search for influenza RNA structure in coding regions to influenza B and C, where conserved and thermodynamically stable regions are predicted to occur at splice sites. The secondary structures of these splice sites are modeled here. The results suggest that influenza RNA splicing may share common structural strategies between the three viral species.

## Methods

### Influenza B and C sequences

The sequences used in this study were obtained from the National Center for Biotechnology Information (NCBI) Influenza Virus Resource [46]. All non-redundant sequences for each segment of influenza B and C were downloaded for the prediction of conserved secondary structure.

### Predicting conserved, thermodynamically stable regions

All non-redundant sequences for segments coding a single protein were translated into amino acid sequences via Seaview 4.3.0 [47,48] and aligned with ClustalW [49]. The aligned sequences were then converted back to nucleotides. Non-redundant sequences of segments that code multiple proteins were aligned according to nucleotides via MAFFT with FFT-NS-i strategy and default parameters [50,51].

Alignments were split into windows of 120 nucleotides (nt) with a step size of 10 nt. Between 6 and 50 sequences, with an average pairwise identity of 80%, were selected for scoring by RNAz 2.1 using the RNAz dinucleotide-shuffling model [39,40]. For a given alignment, RNAz calculates a *z*-score as an estimate of normalized difference in thermodynamic stability of native versus dinucleotide randomized sequences, and a structure conservation index (SCI), which measures the conservation of the minimum free energy of the consensus RNA fold in the alignment. RNAz then uses these as features in a support vector machine (SVM) to output an RNA class probability (p-class), which classifies the RNA fragment as structured or not.

### RNA secondary structure modeling

Five regions within or overlapping RNAz predicted windows with high thermodynamic stability/conservation and/or that contain splice sites were structurally modeled. These regions were extracted as alignments and submitted to RNAalifold [52]. RNAalifold predicts structure via thermodynamic energy minimization [53] coupled with a scoring model for evolutionary conservation. The resulting consensus sequence was also submitted to RNAstructure [54], which utilizes a revised set of nearest neighbor energy parameters to fold single sequences [55]. The minimum free energy (MFE) structure and suboptimal structures [56] were analyzed based on folding free energy and base pairing probability from the calculated base pair partition function [57] and compared to the RNAalifold results. Fragments within these predicted structural regions were extracted for further analysis based on having higher probability pairs (from the partition function) than surrounding structure and on their conservation in the alignment of all non-redundant influenza B or C sequences (paying special attention to evidence of consistent and compensatory mutations). The resulting structural models were used to constrain MC-Fold [58] calculations to suggest possible non-canonical base pairing interactions in predicted loop regions. MC-Fold utilizes high resolution RNA structural information from the Protein Data Bank (PDB) to estimate non-canonical base pairing energies.

DotKnot [59], which folds single sequences, was used to predict pseudoknots in sequences containing splice sites. DotKnot extracts possible stem regions from RNA secondary structure partition function dot plots and assembles pseudoknots according to free energy parameters. Free energies of the pseudoknots were computed with experimentally based thermodynamic energy models [53,60] and loop entropy parameters derived from a diamond lattice model [61,62].

## Results and discussion

The bioinformatics survey for structured RNAs in influenza B and C revealed multiple regions with putative conserved RNA structure (Table 1). Many more high probability prediction windows were identified in influenza C, but these are likely false positives due to the lack of diversity in the input sequences. In several cases there were as few as two influenza C sequences with which to base predictions, versus hundreds in influenza B, and thousands in influenza A. Nevertheless, significant predictions were made in segments 6 and 7 of influenza C, where 28 and 50 sequences were available, respectively. In general, fewer sequence variants for B and C are available versus influenza A due to their lower mutation rate [63-67] and fewer resources for acquiring sequence data for these groups.

**Table 1:** Summary of RNAz scans of predicted structured regions in influenza B and C

	**Segment**	**Protein coded**^**a**^	**Region**	**z-score**^**b**^	**SCI**^**b**^	**p-class**^**b**^
Influenza B	8	NS1, *NEP*	30–220	−1.44 (−2.49)	0.78 (0.86)	0.30 (0.94)
			450–620	−2.27 (−3.29)	0.83 (0.91)	0.81 (0.99)
			720–840	−0.98	0.94	0.37
	7	M1, BM2	0–170	−2.12 (−2.63)	0.66 (0.78)	0.28 (0.56)
	5	NP	1040–1160	−2.29	0.86	0.93
	4	HA	0–160	−2.22 (−3.21)	0.78 (0.87)	0.76 (0.96)
			780–950	−0.81 (−1.4)	0.80 (0.89)	0.20 (0.67)
	3	PA	630–750	−2.19	0.91	0.94
			1870–1990	−2.25	0.66	0.31
			2010–2130	−0.85	0.96	0.46
	2	PB1	650–770	−1.67	0.81	0.39
			1100–1220	−0.57	0.97	0.31
	1	PB2	210–350	−2.82 (−3.12)	0.68 (0.69)	0.85 (0.94)
			670–810	−1.49 (−1.85)	0.83 (0.86)	0.38 (0.62)
			1320–1450	−2.50 (−2.51)	0.76 (0.81)	0.77 (0.91)
Influenza C	7	NS1, *NEP*	190–400	−2.16 (−3.55)	0.70 (0.82)	0.41 (0.99)
			640–800	−1.39 (−2.04)	0.83 (0.87)	0.31 (0.79)
	6	*M1*, CM2	0–160	−2.12 (−2.87)	0.86 (0.91)	0.72 (0.96)
			160–310	−1.13 (−1.36)	0.92 (0.93)	0.33 (0.54)
			350–470	−0.68	0.99	0.37
			450–680	−1.54 (−2.65)	0.96 (0.99)	0.65 (0.98)
			670–790	−1.05	0.97	0.54
			990–1133	−0.97 (−1.19)	0.98	0.54 (0.71)

^a^Proteins coded via splicing are italicized.

^b^Average values for overlapping windows with p-class <0.3. When the region is defined by more than one 120 nt window and the most favorable value for a 120 nt window differs from the average value of overlapping windows, it is presented in parentheses.

**Figure 1:**
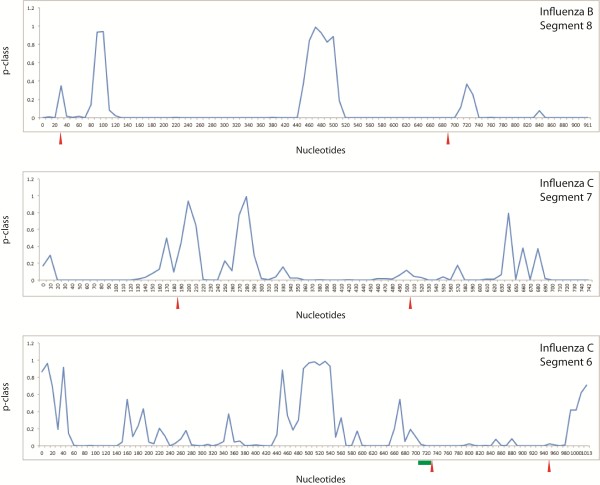
**Plots of p-class for segment 8 of influenza B and for segments 7 and 6 of influenza C.** P-class for 120 nucleotide windows with start position denoted on x-axis. The red arrows indicate splice sites and green box indicates AUG codons.

Similar to results for influenza A [37], where predicted conserved structure appears at or near splice sites, influenza B and C splice sites show evidence for having stable and/or conserved RNA secondary structure (Figure 1). Structural modeling in these regions reveals RNA structures with similarities between influenza A, B, and C, suggesting common strategies for regulation of splicing. In influenza C segment 7, the region near the 3′ splice site is not predicted to have strong structure (p-class range of 0.01-0.12), but a pseudoknot is predicted to occur in this region using the DotKnot program, which is a type of motif forbidden in the RNAz folding algorithm.

### Structures predicted at 5′ splice sites of NEP mRNA in influenza B and C

**Figure 2:**
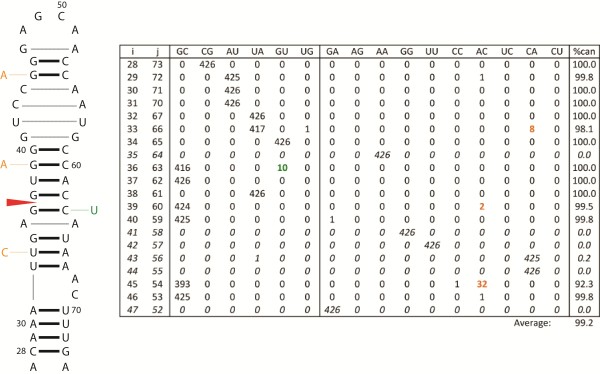
**Secondary structure predicted for the consensus sequence at the 5′ splice site of influenza B segment 8.** Base pair counts from an alignment of all available unique sequences are tabulated to the right of the structure. Base pairing positions (i-j) and types are given in the table with canonical pairs to the left and non-canonical pairs to the right. “%can” column represents the percentage of canonical pairs found at each i-j position in the alignment and the average percent conservation of the structure is given below the column. The italicized alignment positions (*i-j*) indicate possible non-canonical base pairing interactions in loop regions predicted with MC-Fold [58]. Mutations from canonical to canonical base pairs are in green and from canonical to non-canonical base pairs are in orange. The mutations are also annotated on the structure. The red arrow in the structure indicates the splice site and the dashed lines indicate possible non-canonical base pairing interactions in loop regions predicted with MC-Fold [58]. The predicted folding free energy, ΔG°_37_, for the consensus hairpin is −12.5 kcal/mol using parameters from RNA structure [54,55].

**Figure 3:**
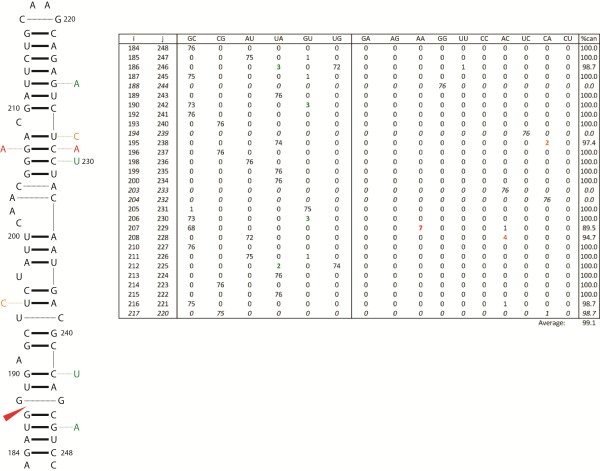
**Predicted secondary structure for the consensus sequence at the 5′ splice site of influenza C segment 7.** Figure annotations and base pair count table are as described in Figure 2. The double point mutation occurring from canonical to non-canonical is indicated in red. The predicted folding free energy, ΔG°_37_, for the consensus hairpin is −22.3 kcal/mol using parameters from RNAstructure [54,55].

Segment 8 in influenza B and segment 7 in influenza C both encode the nuclear export protein, NEP (NS2), involved in vRNP export [68] and in viral transcription and replication regulation [69]. NEP is expressed late in viral infection [8,69]. In influenza B and C, the mature NEP mRNA is generated via alternative splicing [8]. A conserved hairpin is predicted at the 5′ splice site in both influenza B (Figure 2) and C (Figure 3). In each case the 5′ splice site is contained within a helix. Sequestering a splice site in a helical region is a mechanism for regulating splicing [70]. For example, sequestering the 5′ splice site in a helix down-regulates splicing in the hnRNP A1 and SMN2 pre-mRNAs [71,72]. In rat calcitonin/CGRP pre-mRNA, a splice site appears near a 1x1 nucleotide homo-purine internal loop and mutations that change the loop into a Watson-Crick base pair inhibit in vitro splicing [73]; interestingly, in each of the predicted influenza structures containing a 5′ splice site, it also occurs near a homo-purine 1x1 nucleotide internal loop (Figures 2 and 3). Non-Watson-Crick pairs are important in RNA-protein interactions [74] and homo-purine pairs can increase protein binding affinity [75].

These predicted hairpins in influenza B (Figure 2) and C (Figure 3) have <99% base pair conservation. When mutations occurred in stems, they most often were consistent with base pairing. For example, in the nucleotides bordering the splice sites, mutations preserve base pairing: G36-C63 to a G36-U63 in B (Figure 2) and U186-G246 to U186-A246 in C (Figure 3). When mutations in helixes did not maintain canonical pairing, they most often resulted in CA pairs. Protonated C-A^+^ pairs are isosteric with GU pairs and maintain A form helixes [76]. In DNA helixes, C-A^+^ pairs can have pKa’s as high as 7.6 [77] suggesting a similar possibility for RNA. In the 5′ hairpin structure of influenza C segment 7, the A208-U228 pair occurs below the C209 bulge loop and mutates to an AC pair (Figure 3). The flanking GC pair (G207-C229) can mutate to an AA pair. In some contexts, protonated C-A^+^ base pairs adjacent to AA pairs can stabilize internal loops [78] and may be important in RNA-protein interactions [79]. Simultaneous AA (207–229) and AC (208–228) mutations occur in one influenza C sequence (GenBank accession: AB034159).

In contrast to influenza B and C, structures in influenza A are predicted to occur near (79 and 51 nts downstream from), but not overlap with, the 5′ splice sites in segments 7 and 8. Segment 8 of influenza A is homologous to segments 8 and 7 of B and C, respectively, and also produces mRNA for the NEP protein via alternative splicing. The structure in segment 8 of influenza A has been predicted to fold into an extended stem capped by a multibranch [37,45] or hairpin loop, where the hairpin is strongly favored in an avian enriched clade [37]. In vitro mapping experiments, however, reveal a hairpin structure for the consensus sequence that includes the human clade [80]. The 5′ structure in influenza A segment 7 is predicted to form an extended stem topped with a multibranch loop and in vitro mapping is consistent with this structure (Jiang T, Kierzek E, Moss WN, and Turner DH unpublished experiments). Thus, structure is proposed to play roles in splicing of segments 7 and 8 of influenza A [37].

### Structure predicted at the 5′ splice site of influenza C segment 6 contains in-frame start codons

**Figure 4:**
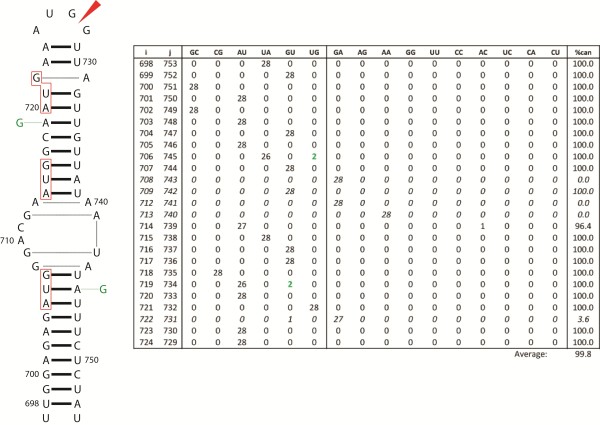
**Secondary structure predicted at 5′ splice site of influenza C segment 6.** Boxed residues indicate cryptic start codons. Other figure annotations and base pair count table are as described in Figure 2. The predicted folding free energy, ΔG°_37_, for the consensus sequence is −13.9 kcal/mol using parameters from RNAstructure [54,55].

A conserved hairpin is also predicted to form in segment 6 of influenza C (Figure 4), which codes for the M1 and P42 (M1′/CM2) proteins. The essential M1 matrix protein of influenza C is produced by splicing of segment 6 mRNA, whereas in influenza A and B, M1 is produced via un-spliced mRNA [81]. The 58 nucleotides surrounding the 5′ splice site of influenza C are predicted to fold into a hairpin, with the 5′ splice site contained within the apical tetra-loop (Figure 4). Splicing elements presented in single-stranded regions of hairpin loops can be more accessible to trans-acting splicing factors [82-85].

CM2 is an ion channel protein, which is also involved in packaging of vRNPs during virus assembly, and release of vRNPs during virus uncoating [86,87]. CM2 is believed to be structurally and functionally equivalent to proteins M2 of influenza A and NB of influenza B [88,89]. CM2 was hypothesized to be produced by one of the three in-frame AUG start codons (Figure 4), especially the start codon at position 705–707 that occurs within a predicted strong ribosome initiation site sequence (RNNAUGG) [90]. It was later found that CM2 is instead produced by proteolytic cleavage of an internal signal peptide in the P42 (M1′/CM2) protein [91,92]. The precise mechanism for the lack of translation initiation at these cryptic start sites is unknown. Interestingly, all three AUG codons occur in the helices of the predicted hairpin (Figure 4). Cryptic AUG start codons sequestered in helices are also found in Polio and Coxsackieviruses [93,94]. Translation initiation is commonly reduced when start codons are embedded in RNA secondary structure [95,96], especially when the mRNA folding free energy near the start codon is more favorable than roughly −12 kcal/mol [97]. Notably, the influenza C segment 6 hairpin has a predicted folding free energy of −13.9 kcal/mol at 37°C suggesting this hairpin may suppress the use of these cryptic start codons in addition to influencing splicing of M1.

The hairpin structure has 99.8% base pair conservation, with two consistent mutations (Figure 4); U706-A745 and A719-U734 change to UG and GU, respectively, preserving base pairing.

### Structures predicted at 3′ splice sites

**Figure 5:**
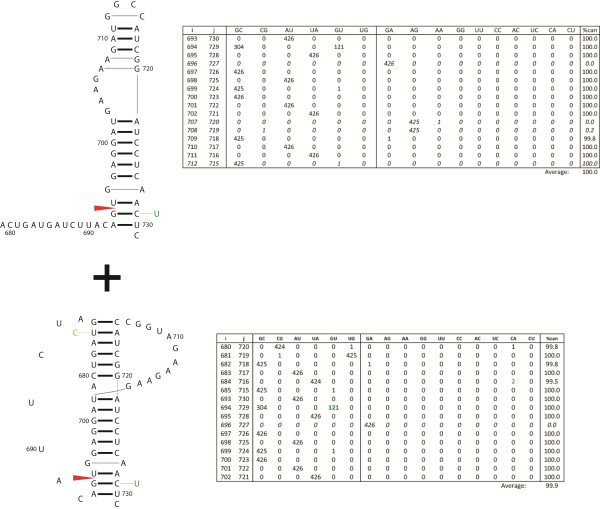
**Secondary structure conformations at the 3′ splice site of influenza B segment 8.** The top structure is the predicted hairpin for the consensus sequence and the corresponding base pair count table. Below is the alternative pseudoknot conformation and base pair count table. These structures were also proposed by Gultyaev et al. [43]. Figure annotations and base pair count table are as described in Figure 2. The predicted folding free energy, ΔG°_37_, for the consensus sequence: in hairpin conformation is −9.3 kcal/mol using parameters from RNAstructure [54,55] and in pseudoknot conformation is −6.0 kcal/mol using parameters from Mathews et al. [55] and Cao and Chen [61].

**Figure 6:**
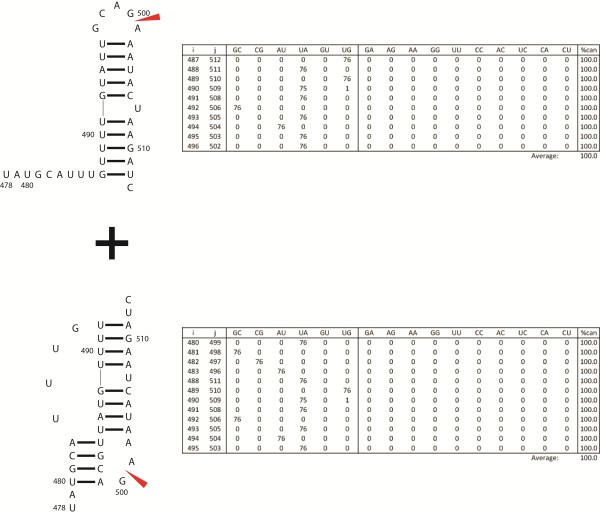
**Predicted secondary structure conformations at the 3′ splice site of influenza C segment 7.** The top structure is the predicted hairpin for the consensus sequence and the corresponding base pair count table. Below is the DotKnot [59] predicted, alternative pseudoknot conformation and base pair count table. Figure annotations and base pair count table are as described in Figure 2. The predicted folding free energy, ΔG°_37_, for the consensus sequence: in hairpin conformation is −3.8 kcal/mol using parameters from RNAstructure [54,55] and in pseudoknot conformation is −9.9 kcal/mol from DotKnot [59].

Stable and conserved structures are predicted for the nucleotides surrounding the 3′ splice sites in segment 8 of influenza B (Figure 5) and segment 7 of influenza C (Figure 6). In segment 8 of influenza B, a 53 nt region including the 3′ splice site can fold into either a hairpin or a pseudoknot [43,44]. In the hairpin (Figure 5), the 3′ splice site occurs in a helical region near a 1x1, homo-purine internal loop, similar to the 5′ splice sites. In the pseudoknot model proposed by Gultyaev et al. [43,44], the 3′ splice site is situated in the loop that spans both helixes. The lower stem can be extended by 4 additional base pairs, which would sequester the splice site and also place it near the 1x1 homo-purine loop (Figure 5). The basal stem encompassing the 3′ splice site is common to both the hairpin and extended pseudoknot models, thus transitioning between the two folds would require a modest structural rearrangement. In segment 7 of influenza C, a 36 nt region encompassing the 3′ splice site is also predicted to fold into a hairpin or a pseudoknot (Figure 6). In the hairpin, the splice site is located in the apical loop and in the pseudoknot it is located in the 3 nt loop joining the two helices.

Three of the 3′ structures in influenza B (Figure 5) and C (Figure 6) are 100% conserved, and the pseudoknot in influenza B is 99.9% conserved. The extended pseudoknot and hairpin folds in segment 8 in B have a frequent consistent mutation, G694-C729 to GU, in the base pair bordering the splice site. The pseudoknotted fold has another single mutation to a non-canonical pair: U684-A716 to a CA pair.

The 3′ splice sites of segments 7 and 8 of influenza A can fold into a hairpin or pseudoknot in a manner similar to that predicted for influenza B and C [37,43,44,98,99]. For segment 7 of influenza A (alternatively spliced to produce M2), native gel analysis showed that the 3′ splice site could form an equilibrium between the pseudoknot and the hairpin [98]. Chemical and enzymatic mapping, as well as oligonucleotide binding support both pseudoknot and hairpin conformations [98]. In the pseudoknot conformation, the 3′ splice site is sequestered in a helix and in the hairpin conformation the splice site is exposed in a 2x2 nucleotide internal loop. The pseudoknot/hairpin conformations predicted at the 3′ splice site of segment 8 were experimentally verified by Gultyaev et al. [43]. These conformational switches are proposed to regulate splicing. A similar switch may regulate splicing of segments 8 and 7 of influenza B and C, respectively.

Influenza B segment 6 and influenza C segment 6 encode ion channel proteins NB and CM2, respectively, but are not predicted to form a pseudoknot/hairpin switch. Unlike M2 in influenza A, NB and CM2 are not produced from mRNA alternative splicing and thus, would not be expected to maintain this structural switch that has apparent importance for splicing regulation.

## Conclusions

This study predicts regions of conserved secondary structure in the coding regions of influenza B and C (+) RNA, which allows comparisons to be made with RNA structures in influenza A. In influenza B and C, regions of high thermodynamic stability and/or base pair conservation are found at splice sites. Similarly, influenza A also has conserved structure at or near splice sites [37,43,45]. In the alternatively spliced influenza A, B, and C RNA segments, structure is predicted at or near the 5′ splice site. In contrast to influenza A, however, the 5′ splice sites in influenza B and C are predicted to be part of hairpins. Four of five 3′ splice sites are predicted to have a pseudoknot/hairpin structural switch. The exception is segment 6 in influenza C. This segment differs from other spliced segments of influenza: it splices to form a UGA stop codon at the splice junction.

Similar to segment 8 of influenza B and segment 7 of influenza C, structure is proposed to occur at the 5′ splice site of influenza C segment 6 (Figure 4). This structure, in addition to containing the 5′ splice site, also buries cryptic start codons in its strong secondary structure. This structural model provides a possible mechanism by which these cryptic start codons are suppressed.

RNA secondary structure is known to play an important role in regulating splicing by hiding or revealing splice sites and protein binding sites, or by changing the distance between regulatory elements [70]. Splicing can also be regulated via protein-induced RNA conformational switching [100,101] or small molecule binding [102,103]. Previous studies have postulated roles for RNA secondary structure in the regulation of splicing in influenza virus [104,105]. The predicted structures in Figures 2, 3, 4, 5 and 6 provide further evidence for the importance of RNA structure in influenza splicing. These results suggest that these RNA structures may be attractive targets for therapeutics as the targeting of RNA splicing with drugs is a growing area of research [106]. Knowing the structure/function relationships of influenza RNAs may be useful in designing therapeutics that specifically target these structures: with small molecules [107-111], oligonucleotides [112-114], or aptamers [115], for example.

## Competing interests

The authors declare no competing interests exist.

## Authors’ contributions

LIDM and WNM designed and conducted computations. LIDM, WNM, and DHT analyzed results. LIDM drafted the majority of the manuscript with contributions from WNM and DHT. All authors have read and approved the final manuscript.
